# Supplementary material for: Plasmodium falciparum-infected erythrocyte co-culture with the monocyte cell line THP-1 does not trigger production of soluble factors reducing brain microvascular barrier function
Source: PLoS One. 2023 May 4;18(5):e0285323. doi: 10.1371/journal.pone.0285323 (PMC10159134; doi:10.1371/journal.pone.0285323)
Supplement: Attachment — Submitted filename: Review Reports by Abebe Fenta.docx [file pone.0285323.s001.docx]

The authors set out “to investigate *Plasmodium falciparum*-infected erythrocyte co-culture with the monocyte cell line THP-1 does not trigger production of soluble factors reducing brain microvascular barrier function”. They co-cultured IT4var14 IE and the monocyte cell line THP-1 for 24 hours and determined whether generated soluble molecules affect barrier function of human brain microvascular endothelial cells. They found that the medium produced after co-culture did not affect endothelial barrier function and similarly no effect was measured after inducing oxidative stress. Anyway, the manuscript can be improved in the following ways:

**ABSTRACT**

It is better, if it includes the **study period,data entry, data collection tool and data analysis methods**

**Itroduction**

**Line 35:** Plasmodium falciparum (P. falciparum), then use the abbreviated one throughout the whole document.

**Line 41:** Nk cells (please use the long fom at the first time), then use the abrivated one throughout the whole document.

**Line 49:** DNA(please write in long form at the 1^st^ time)

**Please do the rest in the same way**

**METHODS**

It is better, if it includes the **study period, data collection tool and study area (Where??? culture done)**

**Please, put the materials and methods after the introduction section not after the discussion section**

**RESULTS:** It is ok

**DISCUSSION:** It is ok

**SUMMARY:** The article is an original and it gives an important clue. But, It needs minor revision.
