# Supplementary material for: Plasmodium falciparum-infected erythrocyte co-culture with the monocyte cell line THP-1 does not trigger production of soluble factors reducing brain microvascular barrier function
Source: PLoS One. 2023 May 4;18(5):e0285323. doi: 10.1371/journal.pone.0285323 (PMC10159134; doi:10.1371/journal.pone.0285323)
Supplement: Attachment — Submitted filename: Response to reviewers.docx [file pone.0285323.s002.docx]

Dr. Janet Storm

Pembroke Place

Liverpool L3 5QA

Janet.Storm@lstmed.ac.uk

Dr. Gebreselassie Demeke

Academic Editor, PLOS ONE

17 April 2023

Dear Dr. Demeke,

*Re: manuscript revision*

We are pleased that our manuscript ‘*Plasmodium falciparum*-infected erythrocyte co-culture with the monocyte cell line THP-1 does not trigger production of soluble factors reducing brain microvascular barrier function’ has been reviewed by PLOS ONE and only requires minor revision.

We thank the reviewer for their comments and below is our point-by-point response, in italic, to their comments and suggestions.

**Abstract**: It is better, if it includes the study period, data entry, data collection tool and data analysis methods.

*Data collection and analysis is written in the materials and methods section, and* w*e have added a sentence at the start of that section with the study period. We do not think it is necessary to add this information to abstract.*

**Introduction**

**Line 35:** Plasmodium falciparum (P. falciparum), then use the abbreviated one throughout the whole document.

*We have written the full name (Plasmodium falciparum) in the abstract and the first time in the introduction and then shortened to P. falciparum*. *It is not common to have P. falciparum in brackets after the full name, it’s not really an abbreviation.*

**Line 41:** Nk cells (please use the long form at the first time), then use the abbreviated one throughout the whole document.

*We now have written the full name (natural killer cells) in the 2 instances it appears in the manuscript.*

**Line 49:** DNA (please write in long form at the 1^st^ time).

*We think this is not needed; it is a universal abbreviation.*

**Please do the rest in the same way**

*In addition to the abbreviations that were already defined in the manuscript, we added the full name, with the abbreviation in brackets, for the following: NLRP3, IL, PBMC, ICAM-1, LPS, SD and SEM. In a few instances we have removed the abbreviation and used the full name when it only appeared once or twice in the manuscript. These are: pathogen-associated molecular pattern, damage associated molecular patterns, glycosylphosphatidylinositol and interferon-gamma.*

**Methods**

It is better, if it includes the study period, data collection tool and study area (Where??? culture done).

*We added a sentence at the start of the materials and methods section that the experiments were conducted at LSTM from January 2019 till October 2021. Data collection is described in each of the methods sections.*

Please, put the materials and methods after the introduction section not after the discussion section.

*We have moved the materials and methods section after the introduction and subsequently adjusted the appearance of the full name/abbreviation in order of the text.*

**RESULTS:** It is ok

**DISCUSSION:** It is ok

**SUMMARY:** The article is an original and it gives an important clue. But, it needs minor revision.

*We are pleased that the reviewer finds our manuscript original and important and has no further requirements to revise our results and discussion sections.*

Yours sincerely,

Dr. Janet Storm
